# Abolition of sagittal T7–T10 dynamics during forced ventilation in AIS patients with Lenke 1A curves

**DOI:** 10.1038/s41598-023-33445-z

**Published:** 2023-04-24

**Authors:** Gonzalo Mariscal, Jesús Burgos, Luis Antón-Rodrigálvarez, Eduardo Hevia, Carlos Barrios

**Affiliations:** 1 School of Doctorate, Valencia Catholic University, Valencia, Spain; 2 Spine Surgery Unit, Hospital Viamed Fuensanta, Madrid, Spain; 3 Division of Paediatric Orthopaedics, Hospital Ramon y Cajal, Madrid, Spain; 4 Spine Surgery Unit, Hospital la Fraternidad-Muprespa, Madrid, Spain; 5 Institute for Research on Musculoskeletal Disorders, Valencia Catholic University, Quevedo, 2, 46001 Valencia, Spain

**Keywords:** Medical research, Outcomes research

## Abstract

In healthy subjects, respiratory maximal volumes are highly dependent on the sagittal range of motion of the T7–T10 segment. In AIS, the abolition of T7–T10 dynamics related to the stiffness induced by the apex region in Lenke IA curves could harm ventilation during maximal breathing. The aim of this study was to analyze the dynamics of the thoracic spine during deep breathing in AIS patients and in healthy matched controls. This is a cross-sectional, case–control study. 20 AIS patients (18 girls, Cobb angle, 54.7 ± 7.9°; Risser 1.35 ± 1.2) and 15 healthy volunteers (11 girls) matched in age (12.5 versus 15.8 years mean age) were included. In AIS curves, the apex was located at T8 (14) and T9 (6). Conventional sagittal radiographs of the whole spine were performed at maximal inspiration and exhalation. The ROM of each spinal thoracic functional segment (T1–T7, T7–T10, T10–T12) and the global T1–T12 ROM were measured. In healthy subjects, the mean T1–T12 ROM during forced breathing was 16.7 ± 3.8. AIS patients showed a T1–T12 ROM of 1.1 ± 1.5 (p < 0.05), indicating a sagittal stiffness of the thoracic spine. A wide T7–T10 ROM (15.3 ± 3.0) was found in healthy controls (91.6% of the T1–T12 ROM). AIS patients showed only 0.4 ± 1.4 ROM at T7–T10 (36.4% of the T1–T12 ROM) (p < 0.001). There was a linear relationship between the magnitude of T7–T10 kyphosis in maximal exhalation and both FVC (% of predicted FVC) and FEV1. In conclusion, Lenke 1A AIS patients show a restriction of the thoracic spine motion with an almost complete abolition of T7–T10 ROM, a crucial segment for deep breathing. T7–T10 stiffness could explain the ventilatory limitations found in AIS patients.

## Introduction

According to the Lenke classification, the type 1 thoracic curve is the most common spinal deformity pattern in adolescent idiopathic scoliosis. Minor proximal thoracic and nonstructural thoracolumbar/lumbar proximal curves may also be present. Type 1 curves in which the line that relates the central sacral vertical line and the apex of the lumbar curve is located between the L5 lumbar pedicles are considered as showing an A lumbar modifier^1^. Surgeries on the thoracic spine are increasingly common. Therefore, knowledge of the thoracic spine’s physiology and its role in pulmonary function seems to be essential^2^.

Restrictive pulmonary impairment caused by a stiffer and less distensible chest wall has been defined as the main pulmonary disorder in scoliosis^3^. Obstruction may also occur in the case of bronchial torsion or associated anomalies^4^. In addition, three anatomical changes in the lungs of scoliosis patients with respiratory involvement were described by Bergofsky et al.^4^: chronic pulmonary emphysema, changes in the small vessels of the lung, and entangled and compressed vessels.

Disorders of the respiratory musculature have been described in patients with kyphoscoliosis (KS) expressed by lower transdiaphragmatic pressures^5^. These alterations lead to a decrease in total lung capacity (TLC) and vital capacity (VC)^6^. Patients with KS have a normal or reduced residual volume (RV). This decrease becomes more evident in thoracic curves and curves greater than 100°^7^. Furthermore, it has been observed that the respiratory system does not respond after the administration of bronchodilators in patients with KS^8^. This finding suggests that additional factors (airway deformity or atelectasis with fluid retention) are responsible for the increase in respiratory resistance.

In KS patients, there are different mechanisms of hypoxemia that can progress to carbonic retention and alveolar hypoventilation^3^. Hypoxemia is mostly due to an alteration of ventilation–perfusion (V/Q). Oxyhemoglobin desaturation leads to increased respiratory muscle weakness, further enhancing hypoxemia^4^. Patients with KS attempt to compensate for respiratory disturbances caused by rib cage deformity with a faster and superficial breathing pattern and increased neural impulses^9^. In addition, KS patients have a poorer tolerance of physical exercise, a situation in which forced ventilation may be crucial^10^. A reliable measure is the 6-min walk test, in which a shorter distance walked than expected was found in patients with KS^11^.

Previous studies have analyzed the movements of the rib cage during respiration^12–14^. Only one study measured angular variations at the level of the thoracic segments in the sagittal plane in healthy patients during deep breathing^15^. Recently, active participation in respiratory function has been attributed to the thoracic spine, particularly to the T7–T10 segment, in healthy subjects^15^. The apex region in Lenke 1A AIS curves usually involves the T7–T10 region.

To our knowledge, no study in the literature addresses the relationship between spinal segmental motion and pulmonary function in patients with scoliosis. Most studies focus on analyzing baseline pulmonary function before and after different surgical procedures. In addition, many studies investigate the respiratory patterns of patients with scoliosis and correlate those with the magnitude of the curve.

We hypothesized that decreased T7–T10 dynamics or ROM assessed by forced inspiration and exhalation in the sagittal plane would reflect the stiffness of the apex region in Lenke 1A curves and could therefore be detrimental to respiratory function in patients with AIS. The confirmation of this hypothesis would provide insights into ventilatory limitations in scoliotic patients and the impact of surgical management on respiratory function in these patients.

The aim of this study was to analyze thoracic spine dynamics by assessing ROM in the sagittal plane of the thoracic spine during deep breathing in patients with AIS and matched healthy controls. Changes in the sagittal profile of the thoracic spine during maximal inspiratory and exhalation movements were measured radiographically in all spinal segments. In addition, lung function was evaluated in AIS patients by forced vital capacity (FVC), expiratory volume (FEV1), FEV1/FVC, inspiratory vital capacity (IVC), and peak expiratory flow (PEF) and related to sagittal thoracic ROM.

## Methods

### Ethical statement

The study protocol was approved by the Institutional Review Board, with protocol number V1-08/05/2016. All the participants were informed about the purpose of the research and were asked to sign a consent form before participating in the study. The procedures were performed in accordance with the ethical standards of the national ethical guidelines for human research ethics and the 2013 revised Declaration of Helsinki. Confidentiality was guaranteed during data collection and subsequent publication of the results.

### Sample size calculation

To compare the means of two populations, sample size was calculated before data acquisition assuming that the standard deviations of both populations are equal with value of 4 Cobb degrees T7–T10 range of motion (the main variable). A significance of 5% and a power of 90% to detect a difference of 5 Cobb degrees was considered. It was also estimated that in each sample 5% of the observations will be lost. The minimum sample size required as a function of the 90% power was 15 participants in each group*.*

### Participants

This was a cross-sectional case–control study. Twenty patients with AIS and 15 healthy volunteers were included after obtaining the appropriate informed consent. The 15 volunteers were age-matched. In AIS curves, the apex was located at T8 (14) and T9 (6). All the 15 volunteers were thoroughly examined to ensure that they had no history of conditions that could affect the study, such as back pain, pulmonary disease, or heart disease. The exclusion criteria were the presence of thoracic vertebral and/or disc changes (previous fractures or Scheuermann disease) and poor-quality radiographic examinations that would have hindered the measurement of all radiological parameters. Table 1 shows the anthropometric characteristics of the sample.

**Table 1:** Age, anthropometric profile, and thoracic curve magnitude of participants.

	AIS patients n = 20	Healthy controls n = 15	p
Age	13.8 ± 1.3 (11.6–15.9)	14.6 ± 1.2	0.068
Female	18 (90%)	11 (73.3%)	0.367^α^
Weight (kg)	46.3 ± 5.8 (43.5–49.1)	55.8 ± 8.7 (51.0–60.6)	0.002
Height (cm)	160.3 ± 8.4 (155.6–164.9)	156.8 ± 6.7 (153.6–160.1)	0.289
BMI	18.8 ± 1.7	21.6 ± 2.1	< 0.001
Cobb (°)	54.8 ± 7.9	NA	

α: Fisher’s exact test.

### Radiographic measurements

Conventional anteroposterior and sagittal functional thoracic radiographs were recorded during the maximal inspiration and exhalation breathing phases. The anteroposterior radiographs were used to identify the number of ribs per participant and to rule out previous vertebral or pulmonary pathologies, while the functional sagittal radiographs were used to measure the different parameters included in this study. In all cases, a previously described standardized procedure for lateral thoracic radiograph acquisition in the standing position was carefully followed by the same radiology technicians^15^. Briefly, the patients were instructed to look straight ahead while keeping their arms raised above shoulder level and their hands holding a support attached to the image detector panel. With the left side of the chest placed against the image receptor, X-rays were centered at the T7 level. The first lateral radiographs were taken at maximal inspiration. The patients were asked to take a deep breath and hold the breath while these images were acquired. Finally, the patients were asked to hold a maximal exhalation for a few seconds so the second lateral radiographs could be taken.

The digital images were measured on a computer using the Surgimap Spine software (Nemaris Inc., Methuen, Massachusetts, USA). Two spine surgeons with significant experience in analyzing the sagittal profile of the thoracic spine on radiographs performed all the measurements. Changes in the angle formed between the proximal and distal endplates of the adjacent vertebral bodies were considered as measurements of the ROM of each functional segment of the thoracic spine. The measurements were compiled in three sectors of the spine (T1–T7, T7–T10, and T10–T12) corresponding to the three anatomical rib attachments. The global ROM of T1–T12 was also registered.

### Pulmonary measurements

The respiratory parameters evaluated were FVC, FEV1, FEV1/FVC, IVC, and PEF. An FVC of less than 80% represents a restrictive pulmonary condition. An FEV1/FVC less than 70% represents a pulmonary obstructive impairment. Respiratory tests were only performed in patients with scoliosis to determine if there was a correlation between ROM restriction and the degree of pulmonary involvement. Since the patients had Lenke 1A classifications and all apexes were located at T8 or T9, only the T7–T10 segment was correlated with respiratory function^15^.

### Statistical analysis

Statistical analyses were performed with the SPSS program, version 21 (IBM Corp., Armonk, New York, USA). As all the measurements comprised continuous numerical data, descriptive results were expressed as mean ± standard deviation (SD). Because of sample limitations, the between-group comparisons of maximal inspiration and exhalation were analyzed using the nonparametric Mann–Whitney U test. Mean changes in kyphosis from inspiration to exhalation in each participant were analyzed with the Wilcoxon rank sum test. Possible correlations between the different radiological measurements and pulmonary function in AIS patients were tested with Pearson’s r test. Results with a p-value < 0.05 were considered statistically significant.

## Results

### Baseline data

Twenty patients with AIS (18 girls, 90.0%; Cobb angle 54.7° ± 7.9°; Risser, 1.35 ± 1.2) and 15 healthy volunteers (11 girls, 73.3%) were included after the corresponding informed consent forms were obtained. The 15 volunteers were age-matched when compared to the patients with AIS (Table 1). The AIS patients had lower body mass and thus lower BMIs than the healthy controls. The AIS curves presented a mean coronal Cobb angle of 54.8° ± 7.9° (maximum 71°, minimum 46°), and the apexes were located at T8 (14) and T9 (6). According to Perdriolle’s method^16^, the mean rotation at the apex level was 17.9° ± 6.8° (maximum 36°, minimum 8°).

### Sagittal range of motion of the spine

**Figure 1:**
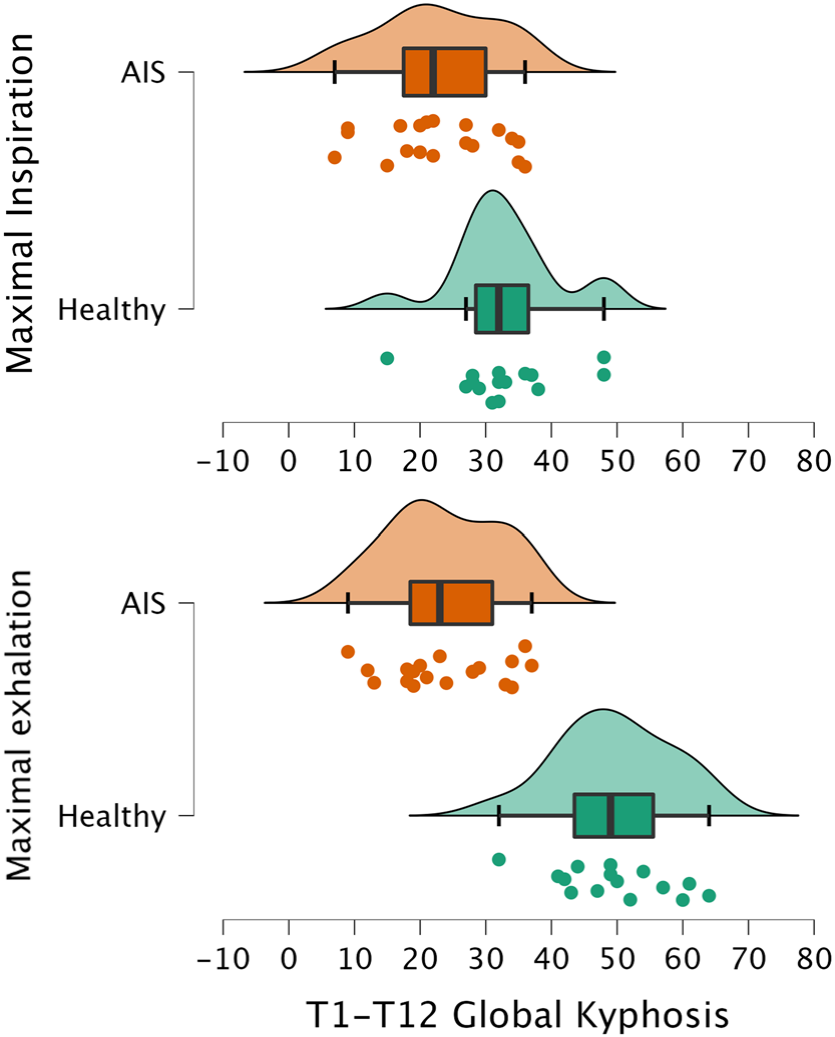
Raincloud plots with horizontal display showing data from the global T1–T12 kyphosis range of motion in the two phases of forced breathing.

Table 2 shows the sagittal ROM of the spine data during forced breathing in both groups of subjects, distinguishing between the different spine segments. In the healthy participants, the total T1–T12 kyphosis increased significantly from maximal inspiration (32.9° ± 8.2°) to maximal exhalation (49.7° ± 8.7°; p < 0.001). In the AIS patients, the total T1–T12 kyphosis showed a short ROM without statistical significance (22.8° ± 9.2° at inspiration and 23.9° ± 8.5° at exhalation) (Fig. 1).

**Table 2:** Sagittal ROM of the different spine segments data during forced breathing in both groups of participants.

Sagittal ROM	Healthy controls		p^α^	AIS		p^α^	Healthy vs AIS	
	Inspiration Mean ± SD (95% CI)	Exhalation Mean ± SD (95% CI)		Inspiration Mean ± SD (95% CI)	Exhalation Mean ± SD (95% CI)		Inspiration p^β^	Exhalation p^β^
T1–T7	19.9 ± 5.2 (16.9–22.7)	22.3 ± 5.2 (19.4–25.2)	0.046*	16.2 ± 5.8 (13.3–18.9)	17.6 ± 5.4 (14.9–20.2)	0.009**	0.068	0.040*
T7–T10	9.9 ± 3.9 (7.7–12.1)	25.3 ± 4.9 (22.5–28.0)	< 0.001**	5.7 ± 4.4 (3.6–7.8)	6.2 ± 4.2 (4.1–8.2)	0.202	0.006**	< 0.001**
T10–T12	3.0 ± 3.1 (1.2–4.7)	2.3 ± 2.2 (1.0–3.5)	0.257	1.1 ± 1.0 (0.5–1.5)	0.4 ± 0.9 (− 0.6–0.8)	0.051	0.061	0.008**
Global T1–T12	32.9 ± 8.2 (28.4–37.4)	49.7 ± 8.7 (44.8–54.5)	< 0.001**	22.8 ± 9.2 (18.4–27.3)	23.9 ± 8.5 (19.8–28.0)	0.005**	0.005**	< 0.001**
T12–S1	60.7 ± 8.1 (56.2–65.1)	69.5 ± 9.1 (64.4–74.5)	< 0.001**	55.1 ± 10.6 (50.1–60.0)	62.6 ± 8.4 (58.0–65.7)	0.001**	0.106	0.031*

α: Wilcoxon Test; β: Man–Whitney U test.

*p < 0.05; **p < 0.001.

**Figure 2:**
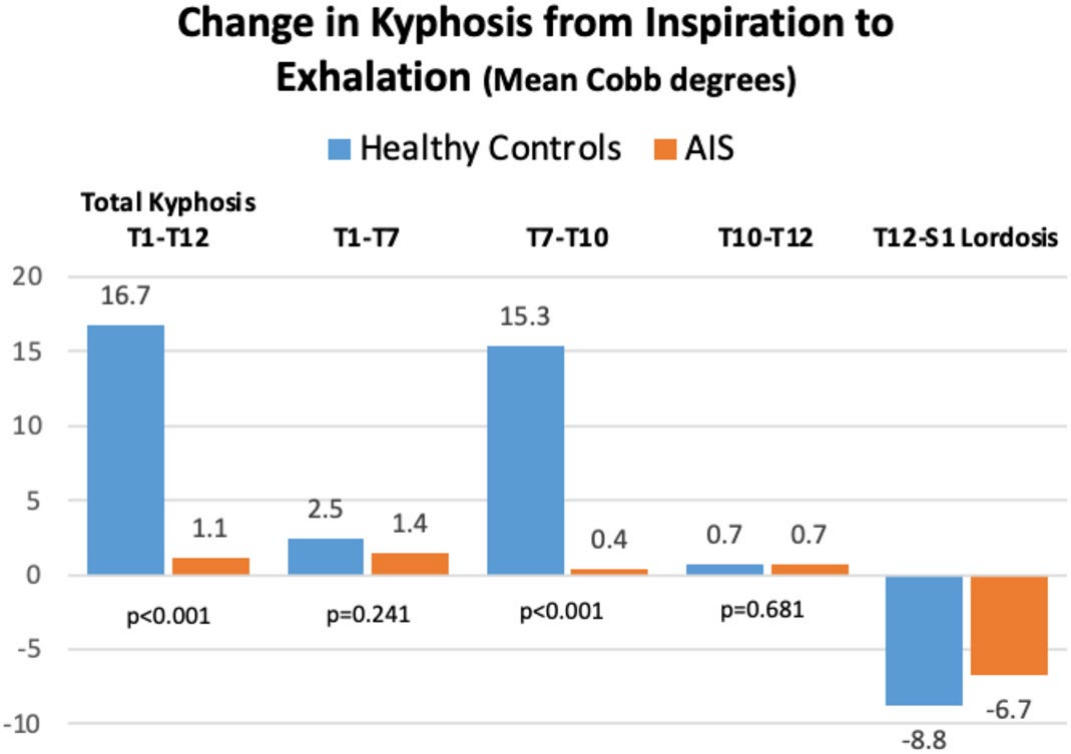
Mean differences in the sagittal range of motion from maximal inspiration to maximal exhalation.

The mean difference from maximal inspiration to exhalation in the T1–T12 physiologic kyphosis was 16.7° ± 3.7° (95% CI 14.8–18.8), reflecting the flexibility of the thoracic spine (50.8%) in healthy adolescents (Fig. 2). The mean difference of the T1–T12 ROM was only 1.1° ± 1.5° (95% CI 0.4–1.8; p < 0.001 as compared to the healthy participants), indicating the sagittal stiffness of the thoracic spine (4.6%) in AIS patients during maximal breathing. There were statistically significant differences between the healthy and AIS participants in the global thoracic kyphosis at both maximal inspiration and exhalation, with lower values in the AIS patients. The data confirms the tendency toward thoracic hypokyphosis in AIS as compared to normal physiologic kyphosis.

**Figure 3:**
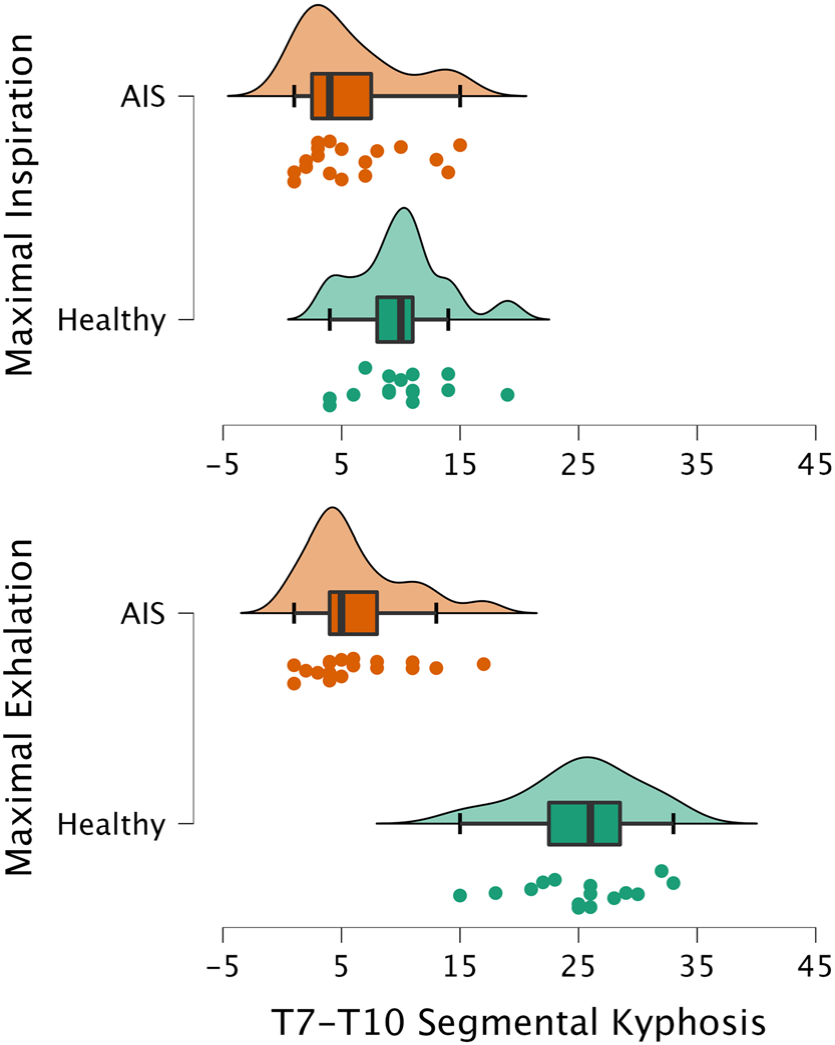
Raincloud plots with horizontal display showing data from the T7–T10 kyphosis range of motion in the two phases of forced breathing.

When the three thoracic segments were analyzed separately, changes in the kyphotic angles of the healthy participants were only apparent in the T7–T10 segment, shifting from 9.9° ± 3.9° in inspiration to 25.3° ± 4.9° (p < 0.001) in exhalation (Table 2). In this segment, the mean change in ROM from inspiration to exhalation was 15.3° ± 2.9°. This change was responsible for 89.8% of the total T1–T12 sagittal movement (Fig. 2). However, the AIS patients showed only 0.4° ± 2.9° of motion at T7–T10 (p < 0.001 as compared to the healthy participants) (Fig. 3). The AIS patients presented a slightly greater restriction of movement in the T1–T7 segment than the healthy controls, without statistically significant changes in the mean differences between inspiration and exhalation kyphotic values (1.4 ± 2.1 in the AIS group vs. 2.4 ± 4.4 in the control group). The T10–T12 segment was stable during deep breathing in both the AIS group and healthy controls (mean differences from inspiration to exhalation: 0.73° ± 1.4° vs. 0.68° ± 2.4°) (Fig. 2).

When the sagittal ROM of the lumbar spine (T12–S1) was evaluated, exhalation was found to induce a reduction in lumbar lordosis in both groups of participants (Table 2). At maximal inspiration, there were no differences between the healthy participants and the AIS patients. However, lumbar lordosis was less pronounced at maximal exhalation (62.6 ± 8.4 vs. 69.5 ± 9.1; p < 0.05). The mean difference from inspiration to exhalation did not differ substantially between the groups (AIS, 6.7° ± 7.2°; healthy controls, 8.6° ± 3.2°) (Fig. 1).

### Pulmonary function of AIS patients

**Figure 4:**
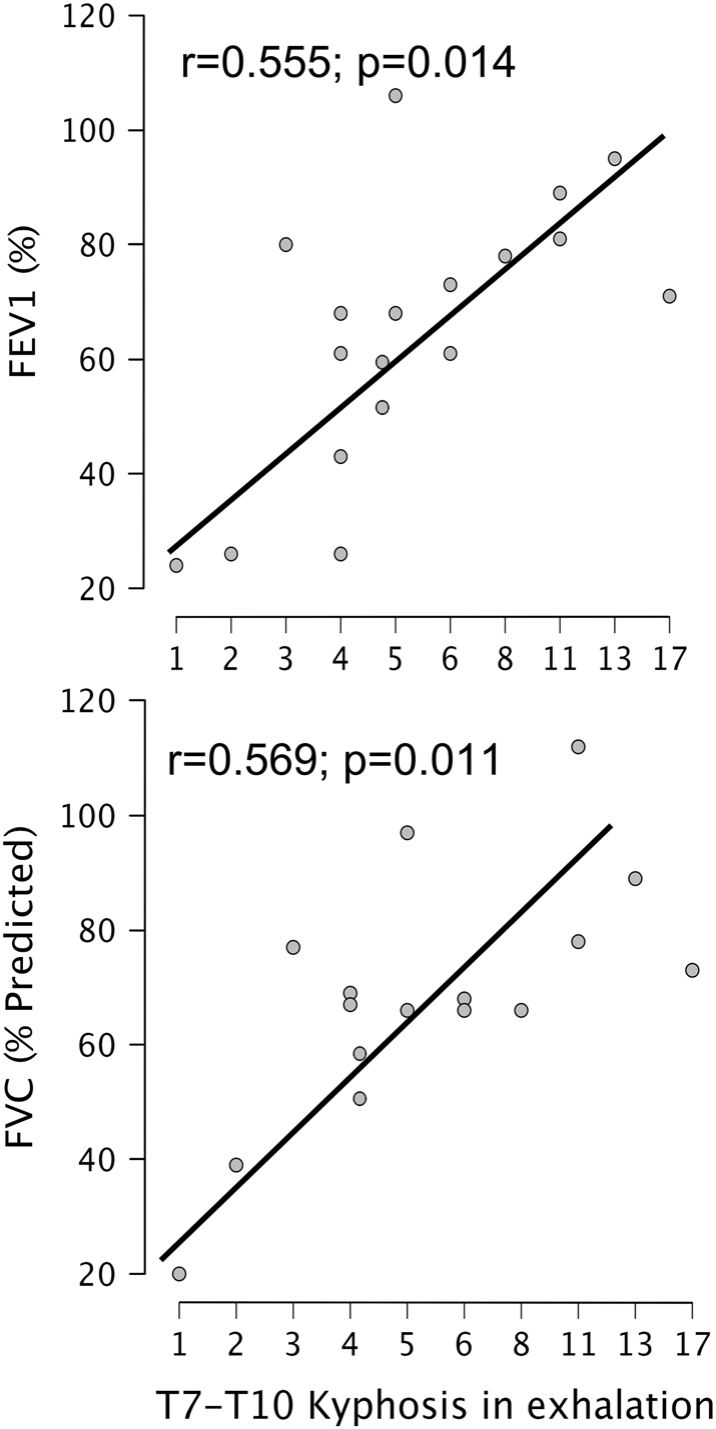
Correlation plots showing the positive interdependence between the magnitude of T7–T10 kyphosis in maximal exhalation and both forced vital capacity (% of precited FVC) and FEV1.

Pulmonary function was analyzed in the AIS patients (Table 3). Out of the 20 patients, 12 (60%) presented a restrictive respiratory pattern assessed by FVC (FVC < 80%). Three AIS patients (15%) showed an additional obstructive pattern (FEV1/FVC < 70%). There was a linear relationship between the magnitude of T7–T10 kyphosis in maximal exhalation and both FVC (% of predicted FVC) and FEV1 (Fig. 4). There was no correlation between pulmonary function and the deformity measured in the coronal plane or the global sagittal ROM of the thoracic spine.

**Table 3:** Pulmonary function in AIS patients.

Respiratory parameters	Mean ± SD	CI 95%
FVC	69.3 ± 21.1	59.4–79.2
FEV1	65.6 ± 24.7	54.0–77.2
FEV1/FVC	94.2 ± 21.1	84.3–104.1
IVC	42.6 ± 12.4	36.8–48.4
PEF	45.9 ± 19.1	37.0–54.8

## Discussion

This work compared spinal ROM during maximal inspiration and exhalation between patients with Lenke-type 1A AIS and healthy subjects. Lung function—as measured by FVC, FEV1, FEV1/FVC, IVC, and PEF—was also analyzed in the AIS group. The results showed a significant decrease in thoracic spine ROM in AIS patients, which was almost entirely attributed to decreased ROM in the T7–T10 segment. The decrease in ROM in this middle segment of the thoracic spine could explain the pulmonary impairment in patients with scoliosis.

The role of paraspinal erector or extensor muscles in the respiratory system is not well established^17, 18^. Their function has been understood to be stabilizing the spine, not contributing to active ventilation^19–21^. However, these muscles play an important role in maximal breathing and are determinants of the segmental movement of the T7–T10 segment during maximal exhalation^21^. An asymmetry in the size of the paraspinal extensor muscle fibers was also observed between the concave and convex sides, although both sides showed some evidence of atrophy^22^. Recent evidence suggests that the ladybird homeobox 1 (LBX1) gene, which encodes the transcription factor ladybird homeobox 1, is involved in AIS development^23^. This gene plays a critical role in guiding embryonic neurogenesis and myogenesis and is vital in determining muscle mass. However, no differences were found in its expression in paraspinal muscles when the convex and concave sides were compared in AIS^23^.

Stiffness of the thoracic and thoracolumbar spines has also been attributed to the abdominal musculature. The abdominal musculature can enhance spinal stiffness, either directly or indirectly, through contraction, which increases intrabdominal pressure (IAP). Linek et al.^24^ demonstrated by shear wave elastography that the external oblique muscle is stiffer on the convex side in right-sided thoracolumbar scoliosis. In idiopathic thoracic and thoracolumbar scoliosis, rotation is a fundamental fact and usually manifests clinically with the presence of a hump. The posterior ribs on the convex side are pushed backward, and the anterior ribs on the concave side are pushed forward. Therefore, the external oblique muscle, because of its attachment to the ribs, may be related to scoliosis. In contrast, in left-sided scoliosis, the transverse muscle on the concave side is stiffer compared with the convex side. In left-sided scoliosis, the vertebrae tend to be rotated to the left; therefore, the transverse processes are positioned more anteriorly. This may explain the greater stiffness of the transverse abdominal muscle on the concave side. The study of vertebral rotation is important for the prognosis and management of patients with scoliosis^25, 26^.

The comparison of concavity and convexity sides in scoliosis is important for understanding respiratory physiology since V/Q disorders depend on the laterality of deformity, with the convex side presenting a better V/Q compared to the concave side, receiving greater compression from the lung parenchyma^27^. An important step would be to study whether there are perfusion disturbances between the different segments in patients with scoliosis. If the T7–T10 segment is stiffer, this area could be responsible for V/Q alterations.

On the other hand, the diaphragm also participates in the stiffness of the spine, especially at the upper lumbar level, given its attachment to L2 and L3. By ultrasound, it has been found that this is a reliable method^28^ to assess the pathological movement of the diaphragm in idiopathic thoracic and thoracolumbar scoliosis^29^. MRI is useful in more accurately assessing the movements of the chest wall and diaphragm in a dynamic and noninvasive way^30^.

The thoracic spine not only has a stabilizing role but also acts as a breathing facilitator by increasing the gas exchange surface in situations that require forced breathing. In this study where maximal breathing was analyzed, both thoracic and abdominal muscles are acting at almost full capacity, and therefore it was not possible to distinguish the role of both groups of muscles in the respiratory function.

Exercise limitation is not uncommon in patients with scoliosis and moderate–severe curves^31^. These types of curves can produce what is known as “ineffective ventilation”: rapid and shallower breathing^3^. On the other hand, in patients with mild curves, the poorer exercise tolerance represented by a reduction in maximal oxygen uptake (VO2max) is due to deconditioning of the limb musculature rather than to the limitation of pulmonary ventilation^31^. The fact that the T7–T10 segment is altered in forced ventilation (e.g., during exercise) could explain the impaired exercise tolerance of these patients.

Episodes of increased forced ventilation requirements are frequent during daily life. The limited ROM of the T7–T10 segment observed in patients with idiopathic thoracic scoliosis with the apex located at this level could be an additional factor involved in the restricted respiratory function of AIS patients. Furthermore, the abolition of T7–T10 motion in the sagittal plane conditioned by the current techniques for spine fusion in AIS requiring surgery could have harmful implications for the respiratory function of these patients.

There is controversy regarding the improvement of pulmonary function after surgery^32^. Previous surgery by thoracoscopy or thoracotomy could create pleural adherences affecting the distensibility of the rib cage^33^. A meta-analysis of different surgical techniques using anterior and posterior approaches in patients with AIS found that the use of an anterior approach did not produce changes in pulmonary function^34^. As for the posterior approach, no differences were found either. Kim et al^33^. observed a significant increase in postoperative lung function after a posterior approach. In contrast, a posterior approach in adolescents requiring multiple segment fixation could be detrimental^34^. This inconsistency in the literature could be due to the heterogeneity of the techniques and patients included in the studies.

To our knowledge, this is the first study to indicate a relationship between the ROM of the different segments of the thoracic spine and pulmonary function in AIS patients. It is also the first study to demonstrate that T7–T10 segment stiffness in patients with AIS could be an associated factor in the deterioration of pulmonary function. This fact is important since extensive spinal fusions involving the T7–T10 segment are currently performed as the unique surgical solution.

This study has some limitations. First, a larger sample size would be desirable, but scoliotic patients are no frequent and additional X-rays exposure, out of the usual clinical controls, is controversial. In addition, the forced breathing maneuvers were voluntary and followed detailed instructions to the participating individuals. However, the maximal strength of the paraspinal muscles during inspiration was never controlled. The standardization of maximal exhalation was also challenging, and both the extension and the flexion of the thoracic spine were influenced by the anthropometric characteristics of the participants. The findings cannot be generalized to other types of scoliosis or to older patients or patients with different vertebral pathologies.

## Conclusion

This study provides new insights into the dynamic behavior of the thoracic spine in patients with AIS during respiratory phases. The results of this study suggest that the stiffness of the thoracic spine could play a relevant role in patients with AIS in situations requiring maximal breathing. Lenke 1A AIS patients showed a restriction of the thoracic spinal range of motion, with almost complete abolition of T7–T10 ROM. T7–T10 stiffness could explain the ventilatory limitations found in AIS patients. Furthermore, the total restriction of T7–T10 after instrumented fusion of the thoracic scoliotic curves could explain the lack of improvement of respiratory function after surgery in AIS patients.

## Data Availability

All data generated or analysed during this study are included in this published article (and its supplementary information files).
